# Diabetic Painful Neuropathy and Restless Legs Syndrome in Diabetes

**DOI:** 10.1007/s13300-018-0376-6

**Published:** 2018-02-09

**Authors:** Sanjay Kalra, Anu Gupta

**Affiliations:** 1 Department of Endocrinology, Bharti Hospital, Karnal, India; 2 Department of Neurology, GB Pant Hospital, New Delhi, India

**Keywords:** Diabetes, Dopaminergic agonists, Gabapentin, Microvascular, Neuropathy, Opioids, Pregabalin

## Abstract

Restless legs syndrome (RLS) and diabetic painful neuropathy (DPN) are two distinct neurological conditions, which share many similarities. As RLS occurs frequently in persons with diabetes, the differential diagnosis and management of RLS and DPN may pose a clinical challenge. This communication describes the etiopathogenesis, clinical features, investigations, and treatment of both conditions. It highlights the similarities and differences between RLS and DPN, and helps the physician plan a rational clinical and therapeutic approach.

## Diabetic Painful Neuropathy

Diabetes mellitus is a syndrome characterized by a myriad of clinical presentations and complications. One important chronic microvascular complication is diabetic neuropathy (DN) [1]. DN is a heterogeneous entity, which includes peripheral sensorimotor and autonomic nervous dysfunction is its ambit. While DN may be asymptomatic, it is often associated with pain. Such DN is termed diabetic painful neuropathy (DPN). The symptomatology of DPN includes a long list of adjectives, which includes intermittent or continuous burning, stabbing, tingling, and numbness, sensation of heat or cold, or itching. The symptoms progress in a distal to proximal distribution, usually starting from the feet [2].

DN is a diagnosis of exclusion, and a label of DN implies that other causes of neuropathy have been excluded. Common causes of neuropathy which must be excluded before a diagnosis of DN is made include alcohol, vitamin B_12_ deficiency, neurotoxic chemotherapy, hypothyroidism, renal disease, malignancies, infections such as human immune deficiency virus (HIV), chronic inflammatory demyelinating neuropathy, inherited neuropathies, and vasculitis [2].

## Restless Legs Syndrome

Restless legs syndrome (RLS) (also known as Willis–Ekbom disease) is a syndrome which is distinct from DPN. However, at the same time, the two diseases share some similarities with each other. RLS is a heterogeneous neurological sensorimotor disease which may impact quality of life significantly. RLS was first described by Willis in 1685. The first diagnostic criteria were laid down by Ekbom in 1945, and have evolved through the decades. Epidemiological studies report that 7–10% of the adult population has symptoms which meet the diagnostic criteria for RLS, and 20–40% of all these report significant suffering. The pathogenesis of RLS has also been explored: low brain iron, low serum iron, peripheral sensory neuropathy, cortical and spinal excitability, hypoxic pathway activation, and genetic factors have been implication in the etiology of RLS [3, 4].

RLS is diagnosed by five essential clinical criteria, which include an urge to move the legs with or without uncomfortable/unpleasant sensations in the legs that begins or worsens at rest/during inactivity, is relieved partially/totally by movement, and worsens during evening/night [3].

RLS is diagnosed after exclusion of medical or behavioral causes such as myalgia, venous stasis, leg edema, arthritis, leg cramps, positional discomfort, and habitual foot tapping. It must be noted that current diagnostic criteria do not list endocrinopathies such as diabetes mellitus and hypothyroidism as exclusion criteria for the diagnosis of RLS. However, secondary RLS [3] is a well-recognized entity, which includes diabetes, hypothyroidism, and kidney failure amongst its etiologies. Thus, the etiopathogenesis of DPN and RLS shows significant overlap. Anemia has a multifaceted relationship with diabetes and metabolic syndrome [5]; DPN is a peripheral sensorimotor neuropathy (similar to RLS); hypoxia and oxidative stress play a role in DPN; and multiple non-glycemic factors contribute to DPN occurrence and progression [6].

RLS has been documented to be more frequent in type 1 diabetes, type 2 diabetes, and in women with a history of gestational diabetes mellitus [7–9]. In a Korean study of 55 children and adolescents with type 1 diabetes (21 males, age 12.6 ± 3.4 years), 13 patients (23.6%, 6 males) met the diagnostic criteria for RLS. Seven of these had a familial history of RLS. A Brazilian case control study of 112 individuals, including 28 with type 2 diabetes, reported a 21.4% prevalence of RLS in type 2 diabetes, as compared to 14.3% in controls (*p* = 0.269). The severity of RLS correlated well with glycemia (*r* = 0.698; *p* = 0.003). An American study of 498 community-dwelling women aged at least 40 years found that 24.5% participants met diagnostic criteria for RLS (17.9% with symptoms at least once per week). After adjustment for various factors, women with a history of gestational diabetes had a higher prevalence of RLS (odds ratio [OR] = 2.7, 95% confidence interval [CI] = 1.3, 5.3) [9].

RLS is also associated with complications of diabetes such as end-stage renal disease and heart failure [10, 11]. RLS is linked to multimorbidity [12] and may suggest poorer metabolic prognosis. Sleep disorders influence glycemic control as well, and inadequate addressal of RLS may contribute to suboptimal diabetes control and outcomes.

This article, which discusses the overlap and dissimilarities between DPN and RLS, is based on previously conducted studies and does not contain any studies with human participants or animals performed by any of the authors.

## Diagnosis of DPN

The objective assessment of DPN is difficult and challenging. Assessment of DPN can be facilitated by tools such as the Michigan Neuropathy Screening Instrument (MNSI) and Neuropathy Disability Score (NDS). The severity of pain is quantified by validated instruments such as the Brief Pain Inventory and Neuropathic Pain Questionnaire (NPQ). Follow-up of persons with DPN can be done using the NPQ and Neuropathic Pain Symptom inventory (Kelly 2005), as well as the Neuro-QoL score [2].

Signs of DPN include impairment of pinprick and temperature sensation (small fiber function), vibration and 10-g monofilament sensation (large fiber function), and loss of ankle reflexes (large fiber function) (Table 1). It must be noted painful neuropathic symptoms are mediated through small nerve fibers, rather than large nerve fibers. Therefore, while quantitative sensory testing (QST) has a role in screening, diagnosis, or follow-up of DPN and risk stratification for foot ulcer, it must be supplemented by a detailed history taking. Skin biopsy (to measure intraepidermal nerve fiber density) and corneal confocal microscopy can be used to assess nerve structure, but their utility is limited to research settings. While skin biopsy can assess small fiber neuropathy, it does not help in diagnosis of DPN. There is no consensus that corneal confocal microscopy can be used as a diagnostic test, especially for DPN. Thus, DPN management is based upon symptom reporting [2].

**Table 1:** Screening and diagnostic tools for diabetic neuropathy

**History**
Positive symptoms, e.g., pain, burning
Negative symptoms, e.g., numbness
Foot ulcer in the past
Suggestive of autonomic neuropathy
**Examination**
Corns, callosities
Foot ulcer/amputation
Stigmata of autonomic neuropathy
**Assessment**
*Protective sensation; large fiber sensation*
10-g monofilament test
*Small fiber sensation*
Pinprick sensation
Temperature sensation
*Large fiber sensation*
Vibration sensation
Ankle reflexes
**Investigations** (rarely needed)
Electrophysiology
Biothesiometry
Confocal microscopy
Skin biopsy

RLS is a clinical diagnosis for which strict diagnostic criteria have been laid down by an international multidisciplinary expert group. Periodic limb movements during sleep (PLMS) or wakefulness (PLMW) are the only sign that can be elicited. RLS, therefore, is characterized by one main symptom: an urge to move the legs, and a single sign: periodic leg movements [3]. No investigations are necessary to screen, diagnose, or monitor RLS.

## Clinical Confusion

Symptoms, in clinical medicine, can be highly elusive and deceptive. A proper history taking depends upon effective two-way communication between patient and physician. Many factors (“good clinical sense”) contribute to the success of this interactive process [13]. The importance of patient–physician communication [14] is especially relevant in conditions such as DPN and RLS, where therapeutic strategies are decided solely on this basis of symptoms. The differences between DPN and RLS are listed in Table 2.

**Table 2:** Differential diagnosis restless legs syndrome vs diabetic painful neuropathy

Parameter	Restless legs syndrome	Diabetic painful neuropathy
Chief complaints		
Chief symptom	Urge to move leg(s)	Pain
Associated symptom	Uncomfortable and unpleasant sensations	Numbness
Epidemiology		
Gender	More frequent in women	No gender gradient
Age	Increase in prevalence with age; seen in children as well	Increases with duration of diabetes
Pregnancy	Common in pregnancy, esp. third trimester	Usually not seen in GDM
Family history	May be present	History of diabetes may be present
Symptoms		
Clinical course	Chronic-persistent or intermittent	Variable/progressive
Common Site	Legs, usually middle of calf or thigh	Legs, usually distal to proximal progression
Symmetry	May be unilateral, bilateral or may change	Usually symmetrical; rarely, may be symmetric
Spread	May spread to arms, other body parts	May spread to arms, with distal to proximal progression
Diurnal variation	More in evening, night May be present throughout the day in severe cases	More in evening, night May be present throughout the day
Association with activity	Sensations began/worsen during rest/inactivity	No such association
Relief	By movement such as walking/stretching/exercise	No such association
Exclusion criteria		
Exclusion criteria	Myalgia, venous stasis, leg edema, arthritis, leg cramps, positional discomfort, habitual foot tapping	Alcohol, vitamin B_12_ deficiency, neurotoxic chemotherapy, hypothyroidism, renal disease, malignancies, infections such as HIV, chronic inflammatory demyelinating neuropathy, inherited neuropathies, vasculitis
Sleep/distress		
Sleep quality/quantity	Impaired	May be impaired
Daytime somnolence	Absent	May be present
Distress	May be significant	May be significant
Signs		
Leg movements	May be reported by bed partner	No such complaint
Periodic leg movements	Present	Absent
Ankle reflex	Present	May be absent
Vibration/pinprick sensation	Normal/may be impaired	Normal
Therapy		
Response to dopaminergic treatment	Positive	Absent
Treatment	Dopamine agonists, alpha-2 delta ligands	Pregabalin, duloxetine, tapentadol, gabapentin

As the symptoms and signs of DPN are non-specific, it is possible that an accurate diagnosis of RLS may be missed in persons with diabetes. This has important clinical implications, as the pharmacotherapy of DPN and RLS differs (Table 3). There are three approved medications for DPN, viz., pregabalin, duloxetine, and tapentadol, though other drugs such as tricyclic antidepressants, gabapentin, venlafaxine, carbamazepine, tramadol, and capsaicin may be used [1]. The management of RLS is pharmacological as well as non-pharmacological. Sleep hygiene, avoidance of caffeine, alcohol, and nicotine, discontinuation of “culprit” medications (SSRIs, diphenhydramine, and dopamine antagonists), and exercise may help in some patients. Anticonvulsants (pregabalin, gabapentin) and dopaminergic agents (pramipexole, ropinirole, rotigotine) are supported by level A evidence for long-term treatment of RLS [15].

**Table 3:** Factors affecting choice of initial therapy in restless legs symptoms

RLS symptomatology	
Associated insomnia Painful restless legs Doubtful diagnosis of RLS vs DPN Severe symptoms Refractory symptoms	α2δ ligand (gabapentin enacarbil) α2δ ligand α2δ ligand Dopaminergic agonist (pramipexole, rotigotine) Oxycodone, naloxone
Comorbid conditions	
Overweight Metabolic syndrome Depression Generalized anxiety disorder Nephropathy Osteoporosis	Dopaminergic agonist Dopaminergic agonist Dopaminergic agonist α2δ ligand/consider iron Ropinirole α2δ ligand

## Refractory Restless Legs Syndrome

RLS is defined as refractory when an individual is unresponsive to monotherapy with maximally tolerated doses of first-line dopamine agonists and alpha-2-delta ligands. Refractoriness can be defined if there is inadequate efficacy, augmentation, or occurrence of adverse effects.

Some causes of refractory RLS include low systemic iron stores (serum ferritin < 75 µg/ml), concomitant use of drugs such as antihistamines, serotonergic antidepressants, and dopamine antagonists, and comorbid conditions like obstructive sleep apnea.

Through this is not mentioned in the current literature, we suggest that diabetes—and DPN in particular—may be a cause of refractory RLS. DPN and RLS may coexist with each other, and inadequate addressal of DPN, including suboptimal glycemic control, may lead to refractoriness of RLS symptomatology. Use of serotonergic antidepressants and dopamine modulators (bromocriptine) in diabetes care may accentuate complaints due to RLS.

## Management of RLS in Diabetes

As RLS and diabetes may occur together, the practicing physician must be aware of the management of RLS. While multiple strategies for the treatment of RLS are available, their choice depends upon various factors. The presence of diabetes is not mentioned as a contributory factor to this decision-making in current guidelines. It must be noted, however, that most clinical trials on RLS list neuropathy, including diabetic neuropathy, as an exclusion criterion.

The two main classes of drugs for RLS are dopaminergic agonists (pramipexole, rotigotine, and ropinirole) and alpha-2-delta ligands (gabapentin enacarbil, pregabalin, and gabapentin). Opioids such as oxycodone and methadone can also be used in patients with refractory RLS. A common cause of secondary RLS is iron deficiency; orally administered or injectable iron must be prescribed if serum ferritin is less than 50–75 µg/ml or transferring saturation is less than 20%. This is especially important in the context of diabetes care, as anemia is a well-known correlate of diabetes, diabetic nephropathy, and metabolic syndrome. Another condition, obstructive sleep apnea, which may contribute to symptoms of RLS, is commonly encountered in persons with diabetes as well.

Table 2 lists some clinical considerations, relevant to diabetology, which may help in initial choice of medication for RLS. Drugs which are used in the management of both RLS and diabetes-related complications include bromocriptine (used earlier for RLS; approved for management of type 2 diabetes), clonidine (used earlier for RLS; used for management of refractory hypertension), and gabapentin (used in both RLS and diabetic neuropathy). Opioids are used in both conditions as well, though pragmatic precautions must be taken while prescribing this class of drugs [16]. On the other hand, drugs used in diabetes care such as serotonergic antidepressants may worsen the symptoms of RLS.

## Summary

All diabetes care professionals must be aware the existence of RLS, its clinical features, and diagnostic criteria.

All diabetes care professionals should be aware of RLS as a potential differential diagnosis and as a possible co-morbidity while evaluating DPN.

All patients being evaluated for DPN should be screened for RLS using updated International Restless Legs Syndrome Study Group (IRL SSG) consensus criteria.

All patients with refractory DPN must be screened for RLS.

All patients with refractory RLS should be screened for diabetes and DPN.

All diabetes care professionals who manage DPN should be able to treat and follow RLS.

Indiscriminate opioid use must be avoided in patients with DPN or RLS.
